# A Case Report on Opioid Withdrawal‐Induced Stress Cardiomyopathy and a Review of the Current Literature

**DOI:** 10.1155/cric/9735423

**Published:** 2026-05-30

**Authors:** Reeju Maharjan, Vidhi Patel, Saba Roa, Khalid Kainat

**Affiliations:** ^1^ Jamaica Hospital Medical Center Internal Medicine Residency Program, New York, New York, USA

## Abstract

Takotsubo cardiomyopathy (TTC), also known as stress‐induced cardiomyopathy or apical ballooning syndrome, is a temporary heart condition characterized by a sudden and reversible weakening of the heart muscle, often triggered by significant emotional or physical stress. First identified in Japan in 1990, the term “Takotsubo” describes the heart′s distinctive shape during this condition, resembling an octopus trap as seen in imaging studies. Opioid withdrawal‐induced stress cardiomyopathy is likely mediated by increased sympathetic activity and catecholamine surge. It may present with acute chest pain, electrocardiographic changes mimicking acute coronary syndrome, modest troponin elevation, and characteristic left ventricular systolic dysfunction with apical or mid‐ventricular ballooning on echocardiography in the absence of obstructive coronary artery disease. Early detection and management with methadone or buprenorphine (an opioid agonist therapy) is important to improve the clinical outcome of the patient. In the context of rising opioid‐related complications, physicians particularly in emergency settings should maintain a high index of suspicion for life threatening conditions. This case report presents an atypical manifestation of Takotsubo cardiomyopathy in the opioid withdrawal population, detailing the patient′s clinical progression, diagnostic evaluation, management, and outcome. Such cases provide valuable insights for clinicians to improve early detection and optimize treatment strategies, ultimately ensuring better patient outcomes.

## 1. Introduction

Stress cardiomyopathy (also called tаkоtѕub*ο* cardiomyopathy or broken heart syndrome) is characterized by temporary and regional systolic dysfunction of the left ventricle (LV), mimicking myocardial infarction (MI), but in the absence of angiographic evidence of obstructive coronary artery disease [1]. The exact pathogenesis of stress cardiomyopathy is not well understood but is strongly linked to catecholamine excess, microvascular dysfunction, and coronary artery spasm [2]. The Mayo Clinical diagnostic criteria includes four criteria that should all be met for diagnosis: transient LV systolic dysfunction (hypokinesis, akinesis, or dyskinesis) on transthoracic echocardiogram (TTE); new electrocardiographic abnormalities (either ST‐segment elevation and/or T wave inversion); or modest elevation in cardiac troponin, absence of obstructive coronary disease/acute plaque rupture and absence of рhе*ο*chr*ο*m*ο*сyt*ο*ma/myocarditis [3]. *Reverse takotsubo syndrome* is a variant of stress cardiomyopathy where hypokinesis of the base with sparing of the mid‐ventricle and apex is seen. This type was present in 2.2% of patients in the International *Τ*аkоtѕub*ο* Registry study [3, 4].

Takotsubo cardiomyopathy and its multiple variants have a varied etiology, although most causes are linked to increased catecholamine surge. One such interesting and emerging cause is opioid withdrawal [5]. Opioid withdrawal mediates sympathetic overdrive and may trigger development of takotsubo syndrome. We present a rare case of opioid withdrawal‐induced stress cardiomyopathy in the form of reverse‐takotsubo cardiomyopathy in a young heroin‐addicted male.

## 2. Case Description

A 25‐year‐old male with a history of opioid use disorder and asthma presented to the emergency department (ED) complaining of abdominal pain, nausea, and vomiting. He disclosed an extensive history of heroin use, consuming eight bags daily over the past 8 months, with his last use in the morning. The patient also reported frequent cannabis use, approximately seven times a week, but denied alcohol consumption or other illicit drug use. Following a stint in prison, where he had been treated with buprenorphine/naloxone, he resumed substance use with synthetic heroin upon his release. He denied experiencing chest pain, dizziness, or shortness of breath but mentioned multiple prior ED visits for opioid withdrawal.

Upon arrival, the patient′s vital signs were largely unremarkable except for a heart rate of 102 beats per minute and elevated blood pressure of 164/106 mmHg. Physical examination findings were unremarkable. Laboratory studies revealed a normal complete blood count (CBC) and comprehensive metabolic panel (CMP). However, troponin levels were markedly elevated, starting at 4.01 ng/mL, peaking at 15.2 ng/mL, and subsequently declining to 12 ng/mL. Electrocardiography (EKG) demonstrated sinus tachycardia with first‐degree heart block and ST‐T wave abnormalities (Figure 1). Urine toxicology was positive only for cannabinoids and negative for other substances, including cocaine, amphetamines, benzodiazepines, methadone, and opiates.

**Figure 1:**
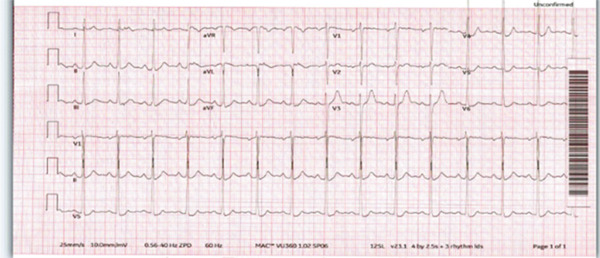
EKG on the day of admission. Normal sinus rhythm. Possible left atrial enlargement. Nonspecific ST abnormality.

Given the elevated troponin levels, cardiology was consulted, and the patient was started on an acute coronary syndrome (ACS) protocol. This included a loading dose of aspirin (325 mg) and clopidogrel (600 mg), followed by maintenance doses of aspirin (81 mg daily), clopidogrel (75 mg daily), and a heparin drip targeting a PTT of 55–75 s.

TTE revealed moderately reduced left ventricular function with hypokinesis of the basal segments and hyperkinesis of the apex, findings consistent with reverse Takotsubo cardiomyopathy. The ejection fraction was reduced to 42%, and there was Grade I diastolic dysfunction (Figures 2 and 3). A viral and respiratory panel was negative, ruling out myocarditis, and a CT coronary angiogram showed no evidence of atherosclerotic coronary artery disease (Figure 4).

**Figure 2:**
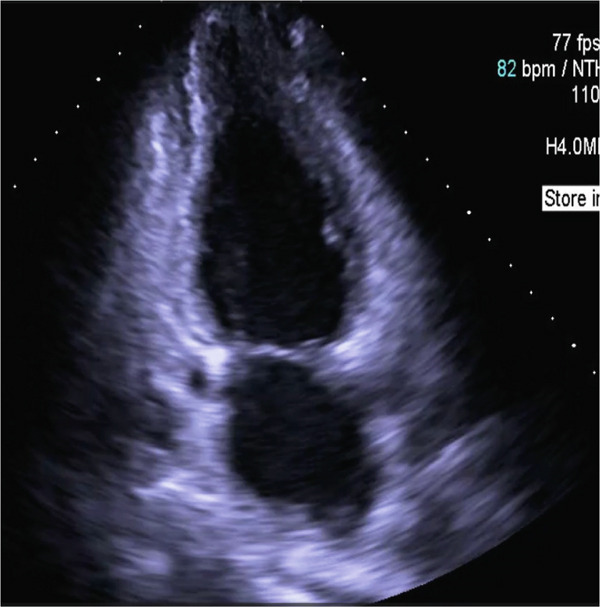
TTE revealed moderately reduced left ventricular function with hypokinesis of the basal segments and hyperkinesis of the apex. The ejection fraction was 42%. Grade I diastolic dysfunction.

**Figure 3:**
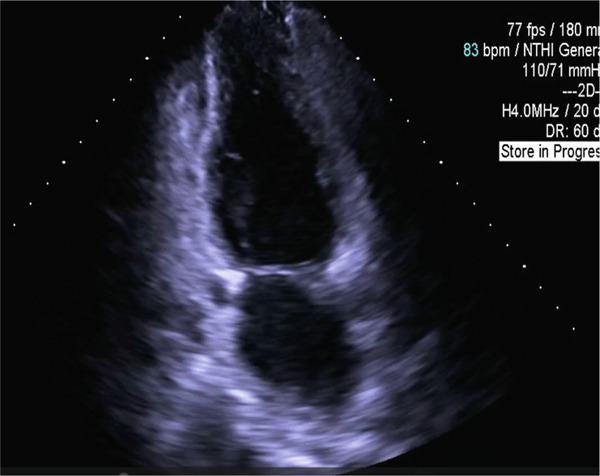
Transthoracic echocardiography (TTE) moderately reduced left ventricular function with hypokinesis of the basal segments and hyperkinesis of the apex, findings consistent with reverse takotsubo cardiomyopathy.

**Figure 4:**
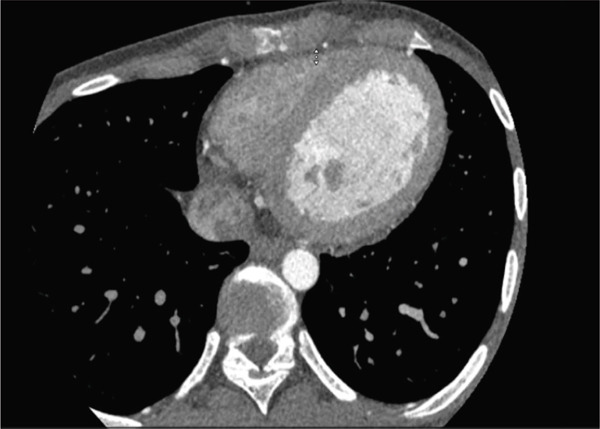
CT angiogram heart 3D image. No evidence of coronary artery disease.

For opioid withdrawal, the patient received two doses of buprenorphine/naloxone in the ED. Following the negative findings of the CT coronary angiogram, ACS medications, including aspirin, clopidogrel, and heparin, were discontinued. Psychiatry was consulted and recommended discharge with buprenorphine/naloxone and follow‐up care for opioid dependence.

Later in the hospital course, the patient developed pleuritic chest pain. A repeat EKG revealed ST‐segment elevation, consistent with pericarditis (Figure 5). Cardiology initiated treatment with ibuprofen (600 mg every 8 h for 2 weeks) and colchicine (0.6 mg twice daily for 3 months). At discharge, the patient was prescribed metoprolol (25 mg daily) and losartan (25 mg daily), as he could not afford sacubitril/valsartan. He was advised to follow up with cardiology within 1–2 weeks and to avoid strenuous physical activity.

**Figure 5:**
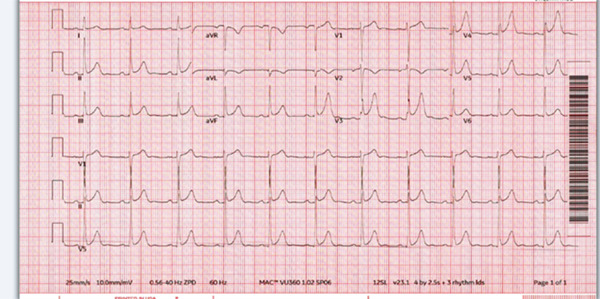
Normal sinus rhythm with ST elevation, early repolarization, pericarditis. Nonspecific T abnormality.

## 3. Follow‐Up and Outcome

Despite discharge recommendations, the patient failed to attend his scheduled cardiology follow‐up appointments. A chart review revealed multiple subsequent ED visits over the following months for opioid withdrawal. These visits highlighted the ongoing challenges in managing both his substance use disorder and cardiovascular condition.

## 4. Discussion

Stress cardiomyopathy is a rare but recognized major adverse cardiac event of acute opioid withdrawal, with few cases published in literature. The mechanism of this phenomenon is related to an increase in catecholaminergic tone as a result of opioid withdrawal with abrupt increases in myocardial oxygen consumption. The effect is the opposite of acute opioid intoxication, which includes hypotension, orthostasis, syncope, and bradycardia, mainly via agonism of the *μ*‐opioid receptors [6, 7]. In addition, contributing to the pathogenesis of opioid‐withdrawal cardiomyopathy is the *β*‐receptor sensitivity and subsequently myocardial stunning [8]. Although the condition is transient, as once the catecholamine surge settles down, the insult to the cardiac myocytes is relieved, it is often life threatening with extremely rare reported cases of acute heart failure, mitral regurgitation, life‐threatening ventricular arrhythmias, or apical LV thrombus [7].

Interesting to note is that the typical stress cardiomyopathy, also known as takotsubo cardiomyopathy or “Broken heart syndrome,” is commonly seen in women. It is reported mostly in postmenopausal women who are under intense emotional stress. As per the international registry of takotsubo cardiomyopathy, 89.8% were women [4]. It is possible that drug‐induced stress cardiomyopathy, especially opioid withdrawal‐induced cases, are seen more commonly in men [8]. This is possibly due to the higher incidence of opioid use disorder amongst the male population, as reported by National Institute on Drug Abuse (NIDA).

Furthermore, reverse takotsubo cardiomyopathy is a variant of takotsubo cardiomyopathy and involves hypokinesis of the base with sparing of the mid‐ventricle and apex. It is only seen in 2.2% of patients in the International *Τ*аkоtѕub*ο* Registry study. [3, 4] Reverse takotsubo cardiomyopathy is seen more commonly in younger women than traditional takotsubo cardiomyopathy, with a mean age of 36 years [9]. However, reverse Takotsubo pattern more commonly seen in drug‐induced types of stress cardiomyopathy [10].

There is one reported case of opioid withdrawal induced reverse takotsubo cardiomyopathy, although it is in a young female [11, 12]. Our case is the first reported case of reverse takotsubo cardiomyopathy due to opioid withdrawal in a male patient.

Takotsubo cardiomyopathy can be triggered by opioid withdrawal. The importance of a multidisciplinary approach of care is evident from this case. Opioid withdrawal can put the body and the heart in significant stress and the combined challenges of managing the withdrawal symptoms and the cardiac dysfunction require coordinated input from various specialists. In this case, the multidisciplinary approach involves, cardiologist to manage the cardiac aspects of TTC, addiction specialist to oversee safe opioid detoxification, and psychiatrist to address to the psychological and emotional stressors contributing to both the withdrawal and the onset of TTC. This collative care model ensures holistic and comprehensive treatment, reduces the risk of complications, and support the patient/s recovery from both physical and emotional aspects of opioid withdrawal, ultimately improving clinical outcomes and preventing recurrence of stress induced cardiomyopathy.

## 5. Conclusion

This case underscores the critical role of continuity of care in managing opioid dependence, particularly in patients recently released from incarceration. The patient′s inability to access buprenorphine/naloxone after release led to withdrawal, which subsequently triggered reverse takotsubo cardiomyopathy. This highlights the physiological impact of untreated withdrawal and the importance of scheduled opioid substitution therapy such as with buprenorphine/naloxone.

The lack of proper coordination between correctional facilities and healthcare providers contributed to this preventable complication. Although no standardized guidelines exist for such scenarios, this case emphasizes the need for a multidisciplinary approach involving addiction medicine, psychiatry, and cardiology. Effective withdrawal management, coupled with structured follow‐up care, is essential to mitigate the risks of severe complications such as stress cardiomyopathy. This report highlights the importance of system‐level interventions to ensure safe transitions of care for opioid‐dependent individuals.

## Funding

No funding was received for this manuscript.

## Consent

No written consent has been obtained from the patients as there is no patient‐identifiable data included in this case report/series.

## Conflicts of Interest

The authors declare no conflicts of interest.

## Data Availability

The data that support the findings of this study are available on request from the corresponding author. The data are not publicly available due to privacy or ethical restrictions.
